# Current Progress on Host Antiviral Factor IFITMs

**DOI:** 10.3389/fimmu.2020.543444

**Published:** 2020-11-30

**Authors:** Linzhu Ren, Shouwen Du, Wang Xu, Tiyuan Li, Shipin Wu, Ningyi Jin, Chang Li

**Affiliations:** ^1^Key Lab for Zoonoses Research, Ministry of Education, Jilin Provincial Key Laboratory of Animal Embryo Engineering, College of Animal Sciences, Jilin University, Changchun, China; ^2^Research Unit of Key Technologies for Prevention and Control of Virus Zoonoses, Chinese Academy of Medical Sciences, Academy of Military Medical Sciences, Changchun, China; ^3^Department of Infectious Diseases, Shenzhen People's Hospital, Second Clinical Hospital of Jinan University, Shenzhen, China

**Keywords:** interferon-inducible transmembrane proteins, entry, virus, interaction, host antiviral factor

## Abstract

Host antiviral factor interferon-induced transmembrane proteins (IFITMs) are a kind of small-molecule transmembrane proteins induced by interferon. Their broad-spectrum antiviral activity and unique ability to inhibit viral invasion have made them a hot molecule in antiviral research in recent years. Since the first demonstration of their natural ability to resist viral infection in 1996, IFITMs have been reported to limit a variety of viral infections, including some major pathogens that seriously endanger human health and social stability, such as influenza A, Ebol, severe acute respiratory syndrome, AIDS, and Zika viruses, etc. Studies show that IFITMs mainly exert antiviral activity during virus entry, specifically interfering with the fusion of the envelope and the endosome membrane or forming fusion micropores to block the virus from entering the cytoplasm. However, their specific mechanism is still unclear. This article mainly reviews the research progress in the structure, evolution, function, and mechanism of IFITMs, which may provide a theoretical basis for clarifying the molecular mechanism of interaction between the molecules and viruses and the research and development of new antiviral drugs based on IFITMs.

## Introduction

Human interferon-induced transmembrane proteins (IFITMs), first reported in 1984, are proteins that can be induced by interferon (IFN). Twelve years later, Alber et al. discovered that these proteins promote antiviral activity (1), providing clues for studying their role in the host antiviral response. In 2009, Brass and colleagues also found that IFITMs were effective limiting factors for the influenza A virus (IAV), further confirming the IFITM antiviral function (2). Since then, research on the interaction between IFITMs and viruses has rapidly become a research hot spot in related fields.

It is reported that IFITM proteins could significantly inhibit IAV, West Nile virus (WNV), Ebola virus (EBOV), SARS coronavirus (SARS-CoV), vesicular pharyngitis virus (VSV), Rift Valley fever virus (RVFV), dengue virus (DENV), Semliki forest virus (SFV), Zika virus, Respiratory syncytial virus (RSV), human immunodeficiency virus-1 (HIV-1), hepatitis C virus (HCV), Reovirus, and other capsular or noncapsular RNA viruses (2–10). Besides this, IFITM proteins also exhibit antiviral activity against individual DNA viruses (5, 11, 12). For example, IFITM1 inhibits frog iridovirus (RGV) replication by preventing the virus from entering cells (11). IFITM1, 2, and 3 can inhibit the early stage of African swine fever virus (ASFV) infection (5). We previously found that IFITM3 protein restricts vaccinia virus (VACV) infection by interfering with virus binding and entry in a low pH-dependent manner, and VACV can also inhibit IFITM3 translation (12).

To date, the antiviral spectrum of this kind of protein involves more than 20 viruses from 12 families. IFITMs, as a branch of the large family of interferon stimulating genes (ISGs), have become star molecules in antiviral immune responses, especially in the study of ISGs, in recent years due to their broad-spectrum antiviral activity and unique ability to inhibit virus invasion. Numerous groups have continued to reveal that IFITMs have a restrictive effect on many viruses and made progress in their antiviral mechanism. In this article, we aim to review the latest research progress on the structure, localization, function, and mechanism of IFITM proteins, providing a reference for the further exploration of the mechanism of IFITMs and the development of their clinical application.

## Structure and Cellular Location of IFITMs

**Figure 1:**
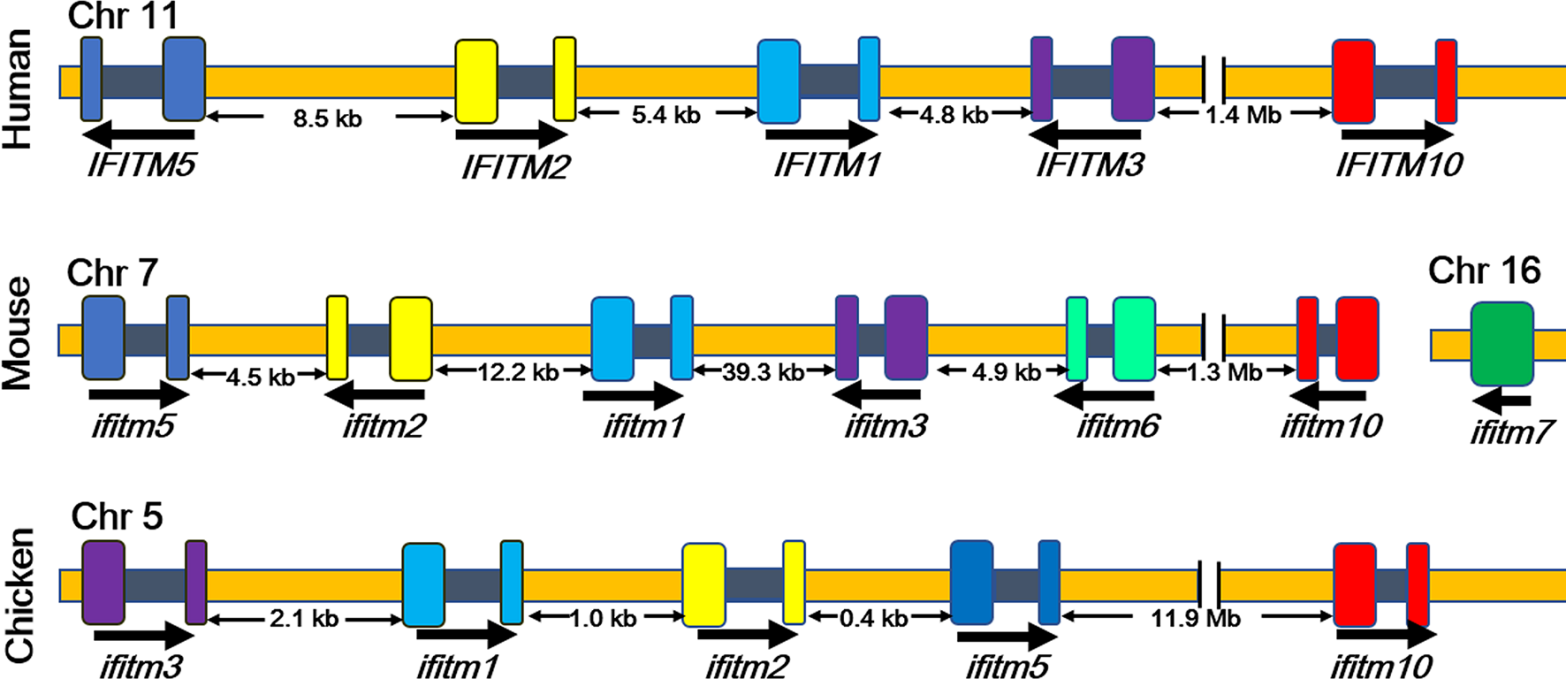
Localization of *ifitm* genes in different species (13). Human *IFITM1*, *IFITM2*, *IFITM3*, *IFITM5*, and *IFITM*10 genes are located on human chromosome 11. Seven *ifitm* genes were found in mice, six of which are located on chromosome 7, and the *ifitm*7 gene is on chromosome 16. Chicken *ifitm1*, *ifitm2*, *ifitm3*, *ifitm5*, and *ifitm*10 genes are located on chromosome 5 of the chicken. Arrows indicate the direction of expression. Exons are expressed in color, and the intron is blank. Chr, chromosome.

IFITMs mainly include the *IFITM1*, *IFITM2*, *IFITM3*, *IFITM5*, and *IFITM*10 genes in humans, and these are located on human chromosome 11 (**Figure 1**) (13–16). IFITM1, IFITM2, and IFITM3 are expressed at low levels in a variety of human tissues, playing roles in embryonic development, cell adhesion, tumorigenesis, and signal transduction as well as antiviral activities (13–15). IFITM5 is only expressed in osteoblasts and participates in bone mineralization and maturation, and the function of IFITM10 is still unknown (17, 18). It was found that the homologous IFITMs also exist in several animals (13–15). For example, five IFITM genes of chickens, including *ifitm1*, *ifitm2*, *ifitm3*, *ifitm5*, and *ifitm*10 genes, are located on chicken chromosome 5 (13–15). There are seven *ifitm* genes in mice, six of which are located on chromosome 7, and the *ifitm*7 gene is on chromosome 16, which is probably reverse-transcribed from the IFITM1 gene (13, 15, 16). Except for the *ifitm7* gene, all IFITM proteins in humans and mice include one intron and two exons.

**Figure 2:**
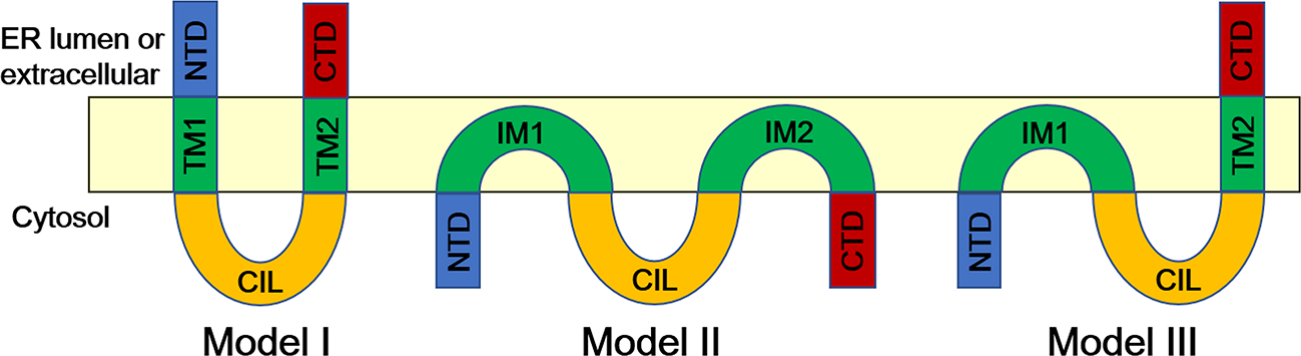
Topological Structure of IFITM Proteins (13). In Model I, IFITMs are U-shaped, two-transmembrane structures with N- and C-terminals either facing the endoplasmic reticulum cavity or extracellular. Another model (Model II) suggests that NTD, CTD, and CIL of IFITM proteins are in the cytoplasm, and IM1 and IM2 are inserted into the lipid bilayer but do not pass through the lipid layer (model II). In the third model (Model III), both NTD and CIL are located in the cytoplasm, and CTD is located in the endoplasmic reticulum cavity. CIL, conserved intracellular loop; CTD, C-terminal domain; IM, intramembrane domain; NTD, N-terminal domain; TM, transmembrane domain.

IFITMs, as transmembrane proteins, can be divided into five domains according to their structural characteristics (**Figure 2**) (6, 15). Human IFITM3 contains a variable and hydrophobic N-terminal domain (NTD, 1-57 aa), a conservative and hydrophobic transmembrane domain (IMD, also known as IM1 or TM1, 58-80 aa), a conservative intracellular cyclic structure (CIL, 81-104 aa), a variable and hydrophobic transmembrane domain (TMD, also known as IM2 or TM2, 105-126 aa), and a short and highly variable C-terminal domain (CTD, 127-133 aa) (6).

To date, there is still controversy about the topological structure of IFITM proteins on the cell membrane, which mainly focuses on the direction of the N- and C-terminals. According to the earliest model, IFITMs have a U-shaped, two-transmembrane structure with N- and C-terminals either facing the endoplasmic reticulum cavity or extracellular (**Figure 2**, Model I) (19). However, Yount et al. proposed another topology structure according to their research results (20). The model suggested that the NTD, CTD, and CIL of IFITM proteins were in the cytoplasm, and IM1 and IM2 were inserted into the lipid bilayer but did not pass through the lipid layer (**Figure 2**, Model II) (20). Bailey et al. reexamined the topological structure of IFITM3 in mice and proved that both NTD and CIL are located in the cytoplasm and CTD is located in the endoplasmic reticulum cavity, thus proposing a structural model of IFITM3 in which NTD is intracellular and CTD is extracellular (**Figure 2**, Model III) (21). Recently, Tian and colleagues further confirmed the Type II transmembrane protein model of IFITM3 by electron paramagnetic resonance (EPR) and nuclear magnetic resonance (NMR) technology and found that IFITM3 contains a C-terminal transmembrane α helix and two N-terminal short intramembrane α helices (22). However, what kind of structure the IFITM protein has remains to be further studied through crystal structure analysis, which helps explore the targets or cofactors that interact with IFITMs, thus explaining their antiviral mechanism.

## Evolution of IFITMs

IFITM proteins belong to the Dispanin protein family, which first appeared as the common ancestor of *Choanoflagellates* and *Metazoa* and then evolved and formed four subfamilies (DSPA to DSPD) in the vertebrates, among which human IFITM1, 2, and 3 with antiviral activity belong to the DSPA subfamily (23).

According to the similarity of the IFITM sequence and its presumed function, it can be further divided into 3 clades. The first clade includes human IFITM1, 2, and 3, which are immune-related IFITMs, and mouse IFITM6 and IFITM7, encoded by intron-free inverse genes derived from the IFITM1 gene. IFITM2 and IFITM3 are highly homologous, and the IFITM1 protein is slightly different. Moreover, many mammals and poultry also have homologs of IFITM1, 2, and 3 although the IFITM2/3-like gene has low homology with human IFITM2 or 3 in other species, such as rhesus monkey. In contrast, clades 2 and 3 consist of IFITM5 and IFITM10, respectively. Although IFITM5 and IFITM10 genes are very close to the sites of human IFITM1, 2, and 3, neither of them can be induced by IFN nor do they have antiviral activity. Therefore, it is assumed that IFITM5 and IFITM10 are reflections of evolution but not positive selection. IFITM5 and IFITM10 also have homologs in many other mammals.

## The Antiviral Spectrum of IFITMs

In 1996, Alber and Staeheli first reported that overexpression of IFITM1 inhibited VSV replication (1). Although this inhibitory effect is not as strong as IFN-induced MxA protein (1), it suggests that IFITM proteins may have an inhibitory effect on viral infection. Besides this, mouse cells overexpressing human IFITM1 are more resistant to VSV infection than control cells, but the effect is not obvious in IAV infection (7). Although this result is different from the current progress (IFITMs have a good inhibitory effect on IAV), it is the first time the antiviral activity of IFITM proteins were described and reported.

In 2009, Brass et al. systematically analyzed and confirmed IFITM3 as a significant limiting factor for IAV infection (2). Further research shows that silencing IFITM3 of U2OS cells can strongly enhance the replication of IAV H1N1 (A/PR/8/34), and siRNA targeting IFITM3 has a significant effect on eliminating IFN-γ mediated virus inhibition (2). We previously found that IFITM3 can suppress H5N1 replication in the early stage of the infection (10). Moreover, overexpression of human IFITM1, 2, or 3 inhibits replication of an IAV H1N1 subtype (A/PR/8/34) and H3N2 subtype (A/Udorn/72) (2, 24, 25). Embryonic fibroblasts (MEFs) from *ifitmDel*^-/-^ mice are more sensitive to IAV than MEFs from wild-type mice, and the anti-IAV effect of type I and II IFN is weakened in *ifitmDel*^-/-^ mice (26). Moreover, the infection of pseudo-retroviruses with different hemagglutinin proteins (H1, H3, H5, and H7) as outer membranes can be effectively inhibited by IFITM1, 2, and 3 (7, 24, 27–29). Studies have shown that arboviruses, including DENV and WNV, have similar sensitivity to IFITM-mediated restriction (30). Before now, it has been found that human IFITMs have potential inhibitory effects on more than 20 viruses in 10 families, mainly inhibiting the entry of viruses. The virus and virus entry characteristics inhibited by IFITMs are shown in **Table 1**. In addition to viruses, IFITM3 can also inhibit *Mycobacterium tuberculosis* invasion (61).

**Table 1:** The viruses that can be inhibited by human IFITMs.

Family/Species	Virus	pH	Inhibitory activity	Endocytic Pathway	Reference
*Alphaviridae*	Semliki Forest virus	pH > 6	IFITM 2/3>1	Clathrin/Dynamin dependent	(31)
	Sindbis virus	Low pH	IFITM 3>2	Clathrin-mediated endocytosis	(31)
*Asfarviridae*	African swine fever virus	acidic pH	IFITM 2/3>1	Dynamin-, clathrin- and cholesterol-dependent endocytosis	(5, 32),
*Bunyaviridae*	Rift valley fever virus	pH 5.5	IFITM 2-3	Caveolin-1 -mediated endocytosis	(33, 34),
	La Crosse virus	pH 5.5	IFITM 1-3	Clathrin-mediated endocytosis	(33)
	Andes virus	pH 5.5	IFITM 1-3	Integrins-, clathrin-, dynamin-, and cholesterol-dependent endocytosis	(33, 35),
	Hantaan virus	pH 5.5	IFITM 1-3	Clathrin-mediated endocytosis	(33, 36),
*Coronaviridae*	SARS coronavirus	pH 4.5	IFITM 1-3	Clathrin-mediated endocytosis	(7, 13, 34, 37),
*Filoviridae*	Marburg virus	pH 4.5	IFITM 1-3	Macropinocytosis	(7, 28, 34),
	Ebola virus	pH 4.5	IFITM 1-3	Macropinocytosis	(7, 13, 34, 38–40),
*Flaviviridae*	Dengue virus	pH 5.5	IFITM 3/1>2	Clathrin-mediated endocytosis	(2, 30, 34, 41),
	West Nile virus	pH 5.5	IFITM 3>1>2	Clathrin-Mediated	(2, 9, 30, 42),
	Yellow fever virus	pH 5.5	IFITM 3>1>2	Clathrin-mediated endocytosis	(2)
	Zika virus	Low Ph	IFITM 3>1	Clathrin-mediated endocytosis	(3, 40, 43, 44),
	Omsk hemorrhagic fever virus	pH 5.5	IFITM 3>1>2	Clathrin-mediated endocytosis	(2)
	*Hepatitis C virus*	pH 6.5	IFITM 1	Clathrin-mediated endocytosis	(34, 45, 46),
	Classical Swine Fever Virus	low pH	IFITM 1-3	Caveola-dependent endocytosis	(47, 48),
*Iridoviridae*	frog iridovirus	low pH	IFITM 1	Caveola-Mediated Endocytosis	(11, 49),
*Orthomyxoviridae*	*Influenza A virus*	*pH 5.5*	IFITM 3>2>1	Clathrin-mediated endocytosis	(2, 34, 40, 50, 51),
*Paramyxoviridae*	Respiratory Syncytial Virus	None	IFITM 1/3	Clathrin-mediated endocytosis	(51–54),
Poxviruses	Vaccinia virus	low pH	IFITM 3	Macropinocytosis	(12, 40),
*Reoviridae*	Reovirus	pH 5.5	IFITM 3	Clathrin-mediated endocytosis	(38, 55),
*Retroviridae*	HIV-1	None	IFITM 1>2/3	Clathrin-dependent endocytosis.	(56–58),
	Jaagsiekte sheep retrovirus	pH > 6	IFITM 1>2/3	Dynamin dependent endocytosis	(24, 34, 59),
*Rhabdoviridae*	Vesicular stomatitis virus	pH 6.5	IFITM 3>1>2	Clathrin-mediated endocytosis	(38, 40, 43, 60),

Unfortunately, IFITMs are not universal antiviral proteins that can resist all viruses. Studies have shown that IFITM proteins have no inhibitory effect on the infection of some viruses, including murine leukemia virus (MLV), arenavirus (LASV), lymphocytic choroid plexus meningitis virus (LCMV), and Crimean-Congo hemorrhagic fever (CCHFV) (7, 25, 62). Warren et al. found that IFITM1, 2, and 3 have no inhibitory effect on human papillomavirus (HPV), cytomegalovirus (HCMV), and adenovirus (Ad5) infected cells (4). Zhao et al. found IFITM proteins promote human coronavirus OC43 infection (63). HCMV utilizes IFITM proteins to facilitate virion assembly compartment formation during infection in MRC5 cells (62). Besides this, *Ifitm3*^-/-^ mice have no obvious susceptibility to bacterial and protozoan pathogens, including *Citrobacter*, *Salmonella typhimurium*, and *Plasmodium berghei*, compared with wild mice (52). Although the reason for these results is still unclear, we can find that most viruses inhibited by IFITMs are enveloped RNA viruses, which mainly rely on low pH to enter cells. Furthermore, the inhibitory activity of IFITMs also has cell and virus specificity.

Besides this, there is still controversy about the effect of IFITM proteins on the *alphavirus*. It was reported that IFITM3 protein expressed *in vitro* had no obvious inhibitory effect on the Chikungunya virus (CHIKV) and Venezuelan equine encephalitis virus (VEEV) (64). However, recent studies show that IFITM2 and IFITM3 proteins can significantly inhibit SFV and Sindbis virus (SINV) although IFITM1 does not affect these viruses (31). *Ifitm3*^–/–^ mice are more sensitive to multiple alphaviruses, including CHIKV, VEEV, SFV, SINV, and O’nyong-nyong virus (ONNV), than wild-type mice, and higher viral loads can be detected in multiple organs of *ifitm3*^–/–^ mice, indicating that IFITM3 can limit the infection and pathogenicity of *alphavirus* (65).

## Antiviral mechanism of IFITMs

### Antiviral Effect of IFITMs Regulated by Post-Translation Modifications

In recent years, progress has been made in the research on the antiviral spectrum, intracellular localization, protein post-translational modification [phosphorylation (66, 67), ubiquitination (21, 68), palmitoylation (21, 69–71) and methylation (66, 72)], and upstream signal pathways generated by IFITMs.

Topological studies of IFITMs indicate that the N-terminal domains of IFITM2 and IFITM3 contain 20 and 21 amino acid residues, respectively, which are crucial for their transport (73). The N-terminal region includes important tyrosine (Tyr, Y), among which Y20 seems to control the cellular distribution of IFITMs (74). It is reported that its antiviral effect decreases or even disappears if the N-terminal of IFITM2 and IFITM3 is deleted or its key amino acid is mutated (74). However, the N-terminal of IFITM1 lacks this domain. Further studies show that IFITM1 is mainly located on the cell membrane and early endosome, and IFITM2 and IFITM3 are mainly located in the endosomal and lysosomal compartments of the endoplasmic reticulum (74), suggesting that the different cell localization of IFITMs determines their antiviral spectrum. Therefore, IFITM1 mainly limits the viruses that fuse to and penetrate the cell surface. For example, IFITM1 is more effective than IFITM2 and IFITM3 in inhibiting Jaagsiekte sheep retrovirus (JSRV) (24). However, IFITM2 and IFITM3 mainly restrict those viruses that enter through the endocytosis as IFITM3 has a stronger inhibition of viral entry by IAV, EBOV, SARS-CoV, etc. (7, 75). Additionally, the cysteine (C71, C72, C105) in IFITM3 can be palmitoylated (69, 70), and the lysine (K24, K83, K88, K104) in its N-terminal and conserved intracellular ring region (CIL) can also be ubiquitinated (20, 56). Codon 70 within the conserved CD225 domain of IFITMs plays a functionally important role in restricting virus entry (70). If this post-translational modification is removed, its antiviral activity is greatly affected (20, 56, 69).

### Possible Antiviral Mechanism of IFITMs

**Figure 3:**
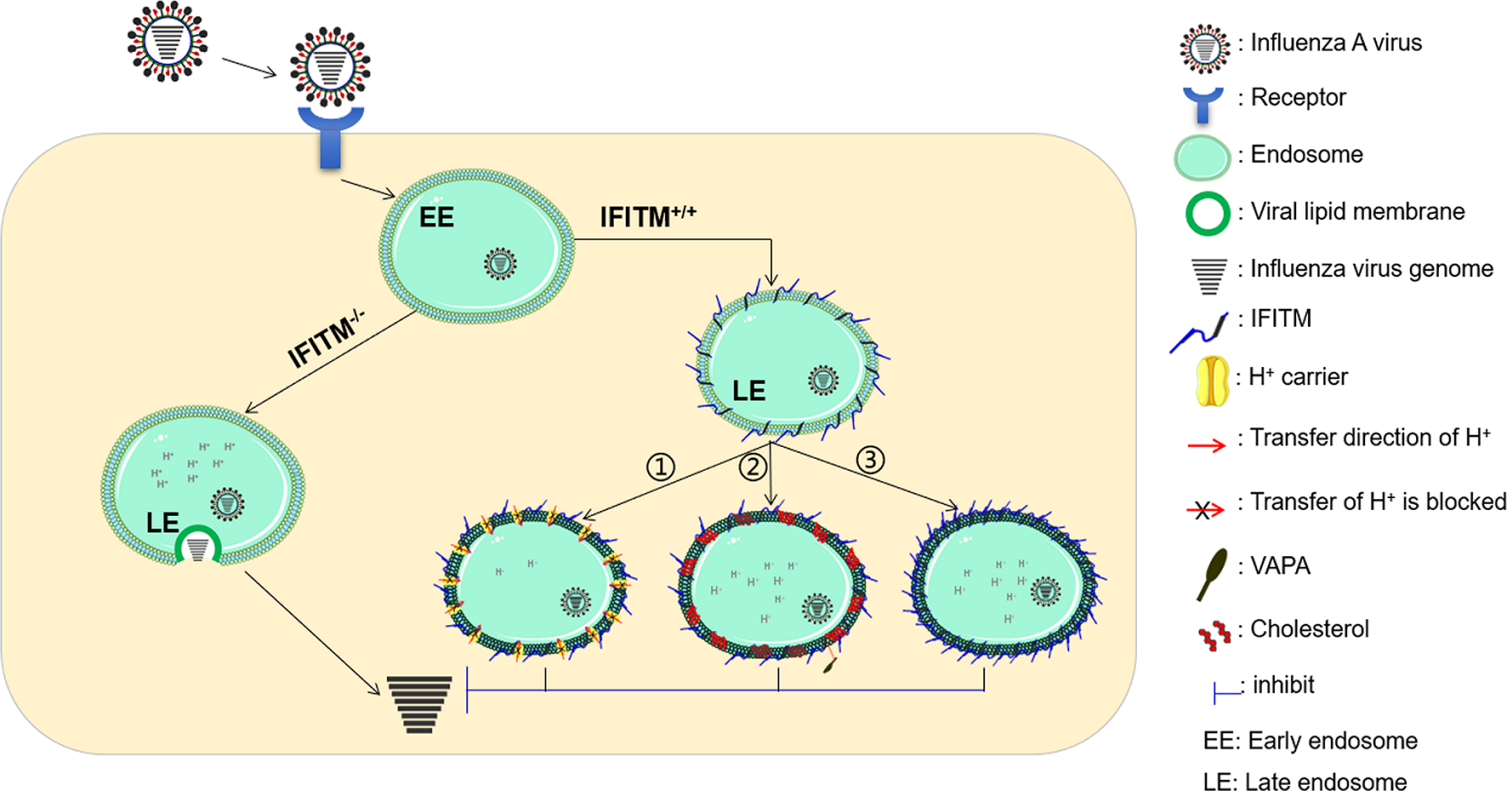
Possible antiviral mechanism of IFITMs. There are mainly three possible antiviral mechanisms. The first possible mechanism is that IFITMs may change the characteristics of the endosomal/lysosomal cavity, making these structures unfavorable for virus fusion (①). Another possible mechanism is that IFITM proteins block the formation of fusion pores following virus-endosome hemifusion by changing the physical properties of cell membranes (②). Besides this, IFITM proteins may also function independently by affecting the cell membrane structure or stimulating effective immune responses and cytokine signaling (③). VAPA, Vesicle-membrane-protein-associated protein A.

To date, IFITM proteins are believed to exert antiviral activity by blocking the fusion of viral membranes, but the research on the molecular mechanism of IFITMs is relatively slow, and the mechanism of IFITMs inhibiting virus entry has not yet been determined. Based on previous studies, there are mainly three possible mechanisms (**Figure 3**).

The first possible mechanism is that IFITMs may change the lysosomes’ characteristics, making these structures unfavorable for virus fusion (13). This mechanism is attractive because it can explain the difficulty of IFITM proteins in inhibiting virus entry in the cytoplasmic membrane. This mechanism is further supported by the results reported by Bailey and colleagues (21). When expressing IFITM3 with N- and C-terminal double tags in cells, it was found that IFITM3 C-terminal tags were cleaved in most cytoplasmic vesicles, but most tags remained in perinuclear vesicles, where the IFITM protein was overexpressed (21). A highly conserved, short amphipathic helix within a hydrophobic region of IFITM3 plays a critical role in IFITM3-dependent inhibition of IAV, Zika virus, VSV, EBOV, HIV, and SARS-CoV-2 infections (43, 71). IFITM3 may accumulate and locate in endosomal vesicles during IAV infection and eventually coat the IAV-containing endosomal vesicles (76). Moreover, IFITM3 can fuse with the incoming virus and enhance the trafficking of the IFITM-virus cargo to the lysosomes for degradation *via* specific S-palmitoylation (76, 77).

Another possible mechanism is that IFITM proteins block the formation of fusion pores following virus-endosome hemifusion by changing the cell membranes’ physical properties (8, 78). Li et al. found that overexpression of IFITM proteins resulted in decreased fluidity of the host cell membranes (24). These results were further confirmed as IFITM1 decreases host-membrane fluidity (79). It was found that IFITM3 interacts with vesicle membrane protein associated protein A (VAPA) and prevents its association with oxysterol-binding protein (OSBP), thereby disrupting intracellular cholesterol homeostasis and inhibiting viral entry (80).

However, other researchers believe that IFITM proteins may also function independently. It is reported that two phenylalanines within IM1 (F75 and F78) of IFITM3 mediate a physical association between IFITM proteins, and the loss of this interaction decreases IFITM3-mediated restriction (41). Further studies show that multiple residues in the NTD and CIL of IFITM3 are required to restrict both IAV and DENV (41). These results suggest that IFITM3 inhibits virus infection by affecting the cell membrane structure (13).

Besides this, IFITM1-3, especially IFITM3, is also expressed in T cells and lymphocytes, which can protect immune cells and the lungs, airway, spleen, skin, and brain from viral infection by stimulating effective immune responses (15). IFITM proteins also affect Th differentiation and are involved in regulating cytokine signaling (15).

### *In Vivo* Function of IFITM3

Researchers infected *ifitm3^–/–^* mice with IAV and found that *ifitm3^–/–^* mice showed higher sensitivity to the virus than wild-type mice with increased lung viral load, aggravated pathological reaction, and decreased CD4^+^ and CD8^+^ T cells and activated NK cells as well as aberrant cardiac electrical activity, increased activation of fibrotic pathways, and fibrotic lesions in the heart (26, 81–83). Wakim et al. found that CD8^+^ resident memory T (T^RM^) cells deposited in the lungs after IAV infection can selectively express IFITM3 protein, facilitating their survival and protection from viral infection during subsequent exposures (84). Everitt et al. found that the viral load of *ifitm3*^–/–^ mice infected with RSV was higher than wild-type mice (52). Diamond and colleagues found that *ifitm3*^–/–^ mice showed greater joint swelling accompanied by higher levels of pro-inflammatory cytokines (such as TNF-α, IL-1β, *etc*.) and viral load during CHIKV infection (65). Besides this, they also found that IFITM3 protein plays a critical role in restricting VEEV and WNV infection and disease progression *in vivo* as *ifitm3*^–/–^ mice were more susceptible to VEEV and WNV infection, resulting in greater virus accumulation in peripheral organs and central nervous system tissues (9, 65). The total number of B cells, CD4^+^ T cells, and antigen-specific CD8^+^ T cells were decreased in WNV-infected *ifitm3*^-/-^ mice compared with that of wild-type mice (9). It is confirmed that the antiviral effect of IFITM3 does not depend on the participation of other IFITMs. These results prove that IFITM3 may limit virus infection through multiple mechanisms, which is of great significance in the occurrence and development of virus-induced diseases.

## Conclusion and Perspective

Viruses must enter cells and use cell components for replication and proliferation to survive, resulting in disease. On the contrary, the host has also evolved mechanisms to prevent virus infection by blocking the virus from entering the host cells. During the last few years, IFITM proteins have been proven to be important proteins for endogenous cell defense against various pathogenic virus infections by blocking virus entry. In particular, IFITM3 protein and its homologs play a direct role in controlling infection of IAV, RSV, and WNV in mice. *In vitro*, experimental data show certain differences in virus types and antiviral activities of IFITM1, IFITM2, and IFITM3 proteins. Therefore, IFITM proteins may inhibit virus infection through various ways or mechanisms that still need to be elucidated. However, the question that cannot be ignored is, how do viruses that are not restricted by IFITMs escape the inhibition of IFITMs, whether they can inhibit IFITMs, and why can viruses that are inhibited by IFITMs not similarly evade this inhibition? Besides this, what is the molecular mechanism of IFITMs inhibiting the virus? What role does the network formed by host IFITMs and viruses play in the interaction between virus and cell, and what specific effects does the network have on the host’s natural immunity and disease process, etc. These mysteries have become the focus of antiviral research, but the ultimate challenge for ISG research, such as that on IFITM proteins, is transforming it into a new strategy for the prevention or treatment of viral diseases.

## Author Contributions

Conceptualization: RL and LC. Writing—original draft preparation: DS and XW. Writing—review, RL, LT, and WS. Figures: DS and RL. Supervision: RL, LC, and JN. Funding acquisition: RL and LC. All authors contributed to the article and approved the submitted version.

## Funding

This work was financially supported by National Natural Science Foundation of China [No 31972719 31702210 31772747], Jilin University Science and Technology Innovative Research Team [JLU-STIRT No. 2017TD-05], the Jilin Province Science and Technology Development Projects [No. 20200402043NC], the Science and Technology Research Program during the 13th Five-year Plan Period of Jilin Educational Committee [No. JJKH20190172KJ].

## Conflict of Interest

The authors declare that the research was conducted in the absence of any commercial or financial relationships that could be construed as a potential conflict of interest.
